# Electrospun Surface-Modified Epidermal Strain Sensors Enable Silent Speech and Hand Gesture Recognition for Virtual Reality Interaction

**DOI:** 10.3390/nano16090520

**Published:** 2026-04-25

**Authors:** Zuowei Wang, Fuzheng Zhang, Qijing Lin, Hongze Ke, Yueming Gao, Wufeng Zhang, Jiawen He, Yan Ma, Na Liu, Dan Xian, Ping Yang, Libo Zhao, Ryutaro Maeda, Yael Hanein, Zhuangde Jiang

**Affiliations:** 1 The State Key Laboratory for Manufacturing Systems Engineering, International Joint Laboratory for Micro/Nano Manufacturing and Measurement Technologies, Xi’an Jiaotong University, Xi’an 710049, China; zwwang2025@foxmail.com (Z.W.); 3507315383@stu.xjtu.edu.cn (H.K.); hejiawen@stu.xjtu.edu.cn (J.H.); my_rock@stu.xjtu.edu.cn (Y.M.); lnbbhqy@stu.xjtu.edu.cn (N.L.); danxian@xjtu.edu.cn (D.X.); ipe@xjtu.edu.cn (P.Y.); libozhao@mail.xjtu.edu.cn (L.Z.); zdjiang@xjtu.edu.cn (Z.J.); 2 The School of Instrument Science and Technology, Shaanxi Innovation Center for Special Sensors and Testing Technology in Extreme Environments, Xi’an Jiaotong University, Xi’an 710049, China; maeda2018@xjtu.edu.cn; 3 The State Key Laboratory for Manufacturing Systems Engineering, Shandong Laboratory of Yantai Advanced Materials and Green Manufacturing, Xi’an Jiaotong University at Yantai, Yantai 265503, China; 4 School of Mechanical and Manufacturing Engineering, Xiamen Institute of Technology, Xiamen 361021, China; 5 Department of Rehabilitation Medicine and Ninth Department of Health Care, The Second Medical Center & National Clinical Research Center for Geriatric Diseases, Chinese PLA General Hospital, Beijing 100853, China; gaoyueming301@163.com; 6 The 214th Institute of China North Industries Group, Suzhou 215163, China; 16606141744@163.com; 7 The Sagol School of Neuroscience, The School of Electrical Engineering, and Tel Aviv University Center for Nanoscience and Nanotechnology, Tel Aviv University, Tel Aviv 69978, Israel; yaelha@tauex.tau.ac.il

**Keywords:** epidermal electronics, assistive communication, wearable sensors, human–machine interface, pattern recognition

## Abstract

Voice disorders severely limit verbal communication, creating a need for intuitive assistive technologies. To meet this need, we present epidermal strain sensors that capture strain signals during silent speech and hand gesture. A thin electrospun nanofiber layer integrated onto commercial polyurethane films guides uniform, controlled microcrack formation in screen-printed carbon conductive paths, achieving a gauge factor up to 243 over 0–40% strain. Signals from the seven-channel strain sensor array are recognized by a hybrid neural network that combines convolutional and Transformer architectures, reaching over 98% accuracy. The recognized outputs are rendered in virtual reality (VR), enabling intuitive, real-time communication. Moreover, the approach simplifies fabrication by enabling crack-based strain sensing with only a thin electrospun surface layer on commercial polyurethane films, eliminating the need for thick freestanding electrospun substrates. This cost-effective approach addresses limitations of conventional electrospun substrates by minimizing the thickness of the electrospun layer, thereby shortening the electrospinning time. Overall, the work demonstrates a method for translating natural non-verbal expressions into speech and text in VR, with promising applications in healthcare and assistive communication.

## 1. Introduction

Voice disorders, often induced by trauma or surgery, significantly limit verbal communication and impose a substantial psychosocial burden [1,2,3,4]. While sign language serves as a primary alternative, it is often ineffective in interactions with individuals unfamiliar with signing. These challenges highlight the need for intuitive communication systems that can capture non-verbal expressions and translate them into speech or text in real time. Beyond voice-related communication, strain variations on the human skin convey rich information about bodily movement and expression [5,6,7]. For example, even when the vocal cords are non-functional due to surgery or trauma, individuals can still express using facial and oral movements in a manner similar to normal speech—this form of expression is referred to as silent speech [8,9,10,11]. Silent speech, driven by facial muscle activity, causes strain variations of the skin that encode linguistic content. Similarly, hand gestures in sign language induce strain variations on the skin of the hands. By attaching flexible strain sensors to face or hand, these non-verbal strain signals can be captured, interpreted, and translated into spoken speech or text outputs.

The acquisition of strain signals from human skin requires a highly sensitive and stretchable strain sensor. Traditionally, metal foil strain sensors have been widely used in fields such as bridge monitoring, civil engineering, and mechanical structures. However, the working mechanism of these metal-based gauges primarily relies on geometric changes induced by tensile deformation, resulting in a relatively low gauge factor (typically ~2) [12,13]. Moreover, commonly used substrates like polyimide (PI) and conductive metals such as copper exhibit poor stretchability, making them unsuitable for conformal and comfortable attachment to the soft, curved surface of human skin to monitor body movement.

To overcome the limitations of traditional metal-based strain sensors, high-sensitivity flexible strain sensors fabricated on stretchable substrates have emerged as a promising solution. Among various structural designs, crack-based strain sensors—in which conductive layers develop microcracks upon stretching—have attracted significant attention [14,15]. During mechanical deformation, the formation and propagation of cracks within the conductive network cause a dramatic increase in electrical resistance, thereby enabling ultrahigh sensitivity. Therefore, fabrication techniques to induce uniform and stable crack formation have become a major research focus for enhancing the reliability and repeatability of crack-based sensors [16,17,18].

Among the fabrication techniques, electrospinning has emerged for producing nanofibrous substrates with excellent stretchability and finely tunable surface morphology. The electrospun surface guides crack formation in conductive layers, optimizing the sensor’s stress–resistance response [19,20].

However, electrospinning-based strain sensors still face challenges in manufacturing efficiency, cost, and scalability. In electrospinning, a low polymer solution flow rate is needed to balance electrostatic forces and surface tension. This low flow rate requires a long production time to form sufficiently thick fibrous films, limiting scalability for large-area applications like skin-mounted sensors [21,22,23].

To address these limitations, we use electrospinning as a surface modification method on commercially available stretchable substrates. Specifically, an ultrathin electrospun nanofiber layer is deposited onto a continuous polyurethane (PU) film. This electrospun layer functions solely as a surface modifier, introducing micro/nanoscale structural features that facilitate controlled crack formation under strain. Consequently, both the electrospinning duration and material consumption are significantly reduced, enabling more efficient and cost-effective production.

In this study, we utilize electrospinning solely as a surface modification technique rather than a means of fabricating the primary substrate. A short-duration electrospinning step introduces finely structured surface topographies on a stretchable commercial PU film, which promotes controlled crack formation under strain. Carbon ink is screen printed on the surface to create crack-induced strain sensors. The resulting sensors demonstrate ultrahigh sensitivity with a gauge factor up to 243 within a strain range of 0–40%.

We further fabricate the sensor into a multi-channel array and attach it to face or hands to collect strain signals from silent speech or hand gesture. Then, a neural network is employed to process the acquired signals, achieving recognition accuracies of 99.5% and 98.75% for hand gesture and silent speech identification, respectively. Additionally, we integrate the recognition outputs into a virtual reality (VR) interface, enabling intuitive, real-time communication in various scenarios. The proposed low-cost, multi-channel, and user-friendly system demonstrates strong potential for healthcare and assistive communication applications.

## 2. Materials and Methods

### 2.1. Sensor Fabrication

The sensor was fabricated using commercial PU films with adhesive backing (HYNAUT Group, medical grade). Electrospinning was applied to the non-adhesive side of the PU film for surface modification. The polymer solution for electrospinning was prepared by dissolving PU particles (BASF 1185A, 100 kDa) in a solvent mixture of N,N-dimethylformamide (DMF) and tetrahydrofuran (THF) at a mass ratio of PU:DMF:THF = 1:2:2. The mixture was stirred magnetically at room temperature until fully dissolved.

Electrospinning was carried out using a TL-Pro-BM system (Tongli Tech). We can not access this URL, please check and revise. The polymer solution was delivered at a flow rate of 0.5 mL/h with a +15 kV voltage applied to the needle. The PU substrate was mounted on a rotating collector with a −5 kV potential for 5 min to collect the nanofibers and form a fibrous surface layer.

Carbon conductive ink (Jujo Chemical) was used to print strain-sensing elements, while stretchable conductive silver ink (Sharex Technology) was used for interconnects. After each printing step, the devices were baked at 120 ^∘^C for 15 min to ensure complete drying and strong adhesion to the substrate.

### 2.2. Characterization

The surface morphology of the electrospun layer and printed carbon paths was examined using scanning electron microscopy (SU8000, Hitachi ). In situ observation of crack formation during tensile deformation was conducted using a laser confocal microscope (LEXT OLS4000, Olympus ).

Resistance–strain characteristics were measured using a source meter (Keithley 2450) while strain was applied with a tensile testing machine (UTM2502, SUNS Technology).

### 2.3. Signal Acquisition in Silent Speech and Hand Gestures

During silent speech and gesture recognition experiments, a custom Arduino-based acquisition circuit was used. The strain sensor was connected in series with a sampling resistor, and the voltage signal was recorded and wirelessly transmitted to a host computer via the built-in Bluetooth module.

### 2.4. Neural Network and VR Display

The recognition algorithm was implemented in PyTorch (https://pytorch.org/) and consisted of two 1D convolutional layers followed by four Transformer encoder layers for feature extraction and recognition. The recognition results were transmitted via socket communication to a Unity 2021-based environment for real-time VR display.

## 3. Results

### 3.1. Sensor Fabrication

**Figure 1:**
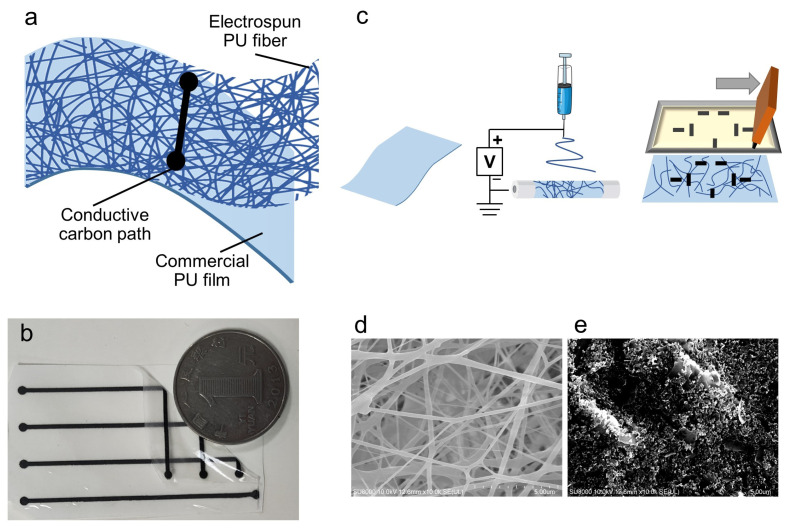
(**a**) Schematic of the strain-sensor architecture. From (**bottom**) to (**top**): a commercial PU film providing mechanical support, a thin electrospun PU layer for surface modification, and carbon conductive traces. (**b**) Photograph of the sensor; one corner is lifted to demonstrate flexibility and transparency, with a coin for scale. (**c**) Fabrication flow: a thin electrospun PU layer is deposited on a commercial PU film as a surface modification, followed by screen printing of the carbon conductive traces. (**d**) SEM micrograph of the electrospun fiber morphology produced by the surface modification. (**e**) SEM micrograph of the carbon conductive traces.

A stretchable, multi-channel strain sensor on a transparent substrate was developed, with the structure illustrated in Figure 1a and the corresponding photograph provided in Figure 1b. The fabrication process involves electrospinning and screen printing, as shown in Figure 1c. First, a commercial polyurethane (PU) film was treated with electrospinning to modify its surface, followed by screen printing of conductive carbon ink to form strain-sensitive conductive paths.

The commercial PU film features a skin-adhesive backing that enables direct attachment to the skin. Electrospinning was carried out for 5 min on the non-adhesive side to deposit a thin layer of PU nanofibers, resulting in a textured surface morphology, as shown in Figure 1d. Direct cross-sectional thickness measurement of the electrospun layer was not feasible, as the fibrous structure suffered from compression and structural damage during sample cutting. We therefore estimated the layer thickness via a calibrated constant deposition rate method: under identical process parameters, 4 h of continuous electrospinning yielded a free-standing thick film with an average thickness of 0.22 mm (expanded uncertainty: 0.01 mm, k = 2, 95% confidence level, *n* = 5). Based on the linear deposition rate, the thickness of the 5 min electrospun surface modification layer in this work is calculated to be 5 μm.

After surface modification, conductive carbon paths were screen-printed, and the surface morphology of carbon paths is shown in Figure 1e. Under strain, the printed paths develop cracks, leading to an increase in resistance and enabling strain detection.

### 3.2. Characterization of the Strain Sensor

To characterize sensor performance and investigate the effect of electrospinning on crack formation under strain, the resistance–strain response, I–V characteristics, along with in situ crack observations were conducted. The results show the electrospun nanofiber surface induced uniform and stable crack formation, yielding a gauge factor up to 243 within 0–40% strain. Moreover, integrating a thin electrospun fiber layer as a surface modification on commercial PU achieved performance identical to an independent thick electrospun film, while reducing the layer thickness and the associated electrospinning time. This cost-effective approach enables high-performance strain sensors.

**Figure 2:**
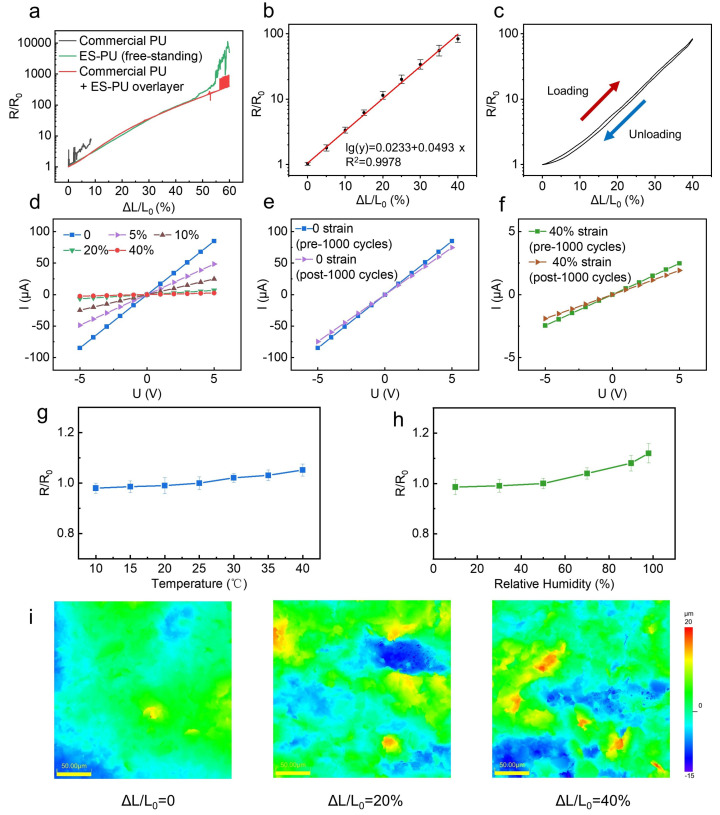
(**a**) Relative resistance–strain responses under tensile loading for three sensors on different substrates: untreated commercial PU film, a fully electrospun (free-standing) PU film, and a commercial PU film modified with a thin electrospun layer. (**b**) Resistance–strain curves with linear fits; error bars represent the standard deviation across five independent devices (*n* = 5). (**c**) Dynamic sensor output during stretching; area between loading/unloading curves indicates hysteresis. (**d**) I–V characteristics at different strains. (**e**) I–V characteristics comparison at 0 strain (pre– v.s. post–1000 stretching cycles). (**f**) I–V characteristics comparison at 40 % strain (pre– v.s. post–1000 stretching cycles). (**g**) Variation of the initial resistance of the sensor at different temperatures (relative humidity fixed at 50%). Error bars are obtained from independent measurements of five devices. (**h**) Variation of the initial resistance of the sensor at different relative humidities (temperature fixed at 25 °C). Error bars are obtained from independent measurements of five devices. (**i**) In situ laser scanning confocal images during stretching. The elevated regions that become progressively discontinuous correspond to the conductive carbon ink, whereas the lower fibrous structures are the exposed electrospun PU fibers.

The change in resistance under tensile strain was measured and compared across three cases: the proposed device, device on commercial PU surface, and a device on a fully electrospun substrate. As shown in Figure 2a, the device fabricated on commercial PU exhibits large and unstable resistance fluctuations at the initial stage of stretching, making it unsuitable for reliable strain measurement. In contrast, the device fabricated on a free-standing electrospun substrate exhibits continuous and stable resistance growth over a wide strain range. The device proposed in this work, in which a thin ES-PU layer was applied to modify the commercial PU film, displays a resistance–strain response and strain range identical to those of the free-standing electrospun device. This indicates that a thin surface electrospun fiber layer is sufficient to induce uniform crack formation. Table 1 provides a detailed comparison of the performance of different sensors in Figure 2a, demonstrating the advantages of electrospinning as a surface modification method in reducing time consumption and improving efficiency.

Further experiments verified the device’s gauge factor (GF), repeatability, hysteresis behavior under dynamic loading–unloading cycles, and linear I–V characteristics [Figure 2b–d]. The GF is defined as the ratio of the relative resistance change to strain; its calculation is given in Equation (1). The proposed sensor achieves a GF up to 243 within the 0–40% strain range and exhibits highly linear I–V curves, facilitating signal readout. The variations in the I–V characteristics of the sensor before and after 1000 stretching cycles are shown in Figure 2e,f. Although the sensor output decreases, it still remains functional.

As shown in Figure 2g,h, the initial resistance of the sensor increases slightly with rising ambient temperature and relative humidity (RH). This trend is consistent with findings from previously reported studies. A plausible mechanism underlying this behavior is outlined below. With increasing temperature, the sensor substrate undergoes thermal expansion. With increasing humidity, the electrospun PU adsorbs ambient water molecules and swells, also leading to substrate expansion. The conductive paths on the substrate possess a lower thermal expansion coefficient and water absorption than the substrate. This mismatch in expansion gives rise to the widening of microcracks within the conductive network, thus increasing the electrical resistance of the sensor [24,25]. For future commercial applications, such environmental interference can be reduced by introducing a passivation layer.

(1) $$\mathrm{GF}=\frac{\Delta R/R_{0}}{\varepsilon }=\frac{R/R_{0}-1}{\Delta L/L_{0}}$$

To visualize crack formation, in situ laser confocal microscopy was performed during stretching, as shown in Figure 2i. At 0 strain, the carbon layer remained continuous, providing low electrical resistance. At 20% strain, cracks are significant, exposing the underlying nanofiber substrate and reducing effective conductive pathways. At 40% strain, cracking intensified, with increased substrate exposure and further reduction of conductive paths. These observations are consistent with the resistance–strain measurements.

### 3.3. Strain Signal Acquisition in Silent Speech and Hand Gesture

**Figure 3:**
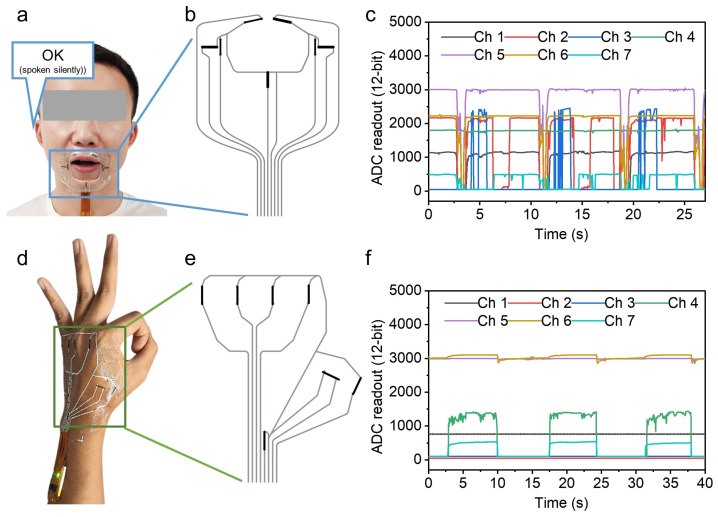
(**a**) A seven-channel strain-sensor array and the data-acquisition circuit applied to the participant’s face during the silent articulation of “OK.” (**b**) Design of the facial strain-sensor array: black traces are screen-printed carbon strain-sensing lines; silver traces are strain-insensitive silver lines. (**c**) Signals acquired during three consecutive silent articulations of “OK.” (**d**) A seven-channel strain-sensor array and data-acquisition circuit applied to the participant’s hand while forming the “OK” hand gesture. (**e**) Design of the hand strain-sensor array: black traces are carbon strain-sensing lines; silver traces are strain-insensitive silver lines. (**f**) Signals acquired as the hand moves from a relaxed posture to the “OK” hand gesture; three consecutive repetitions are shown.

The proposed strain sensors were configured into arrays and applied to specific regions of the face and hand, as shown in Figure 3a,d. These arrays captured resistance changes caused by skin strain during silent speech and hand gesture. The sensor arrays designed for silent speech and hand gesture recognition are shown in Figure 3b,e. On electrospun-treated substrates, seven conductive carbon paths were patterned via screen printing. Stretchable soft silver ink, with much lower square resistance, was printed to connect the carbon paths to pins for signal readout. The silver ink maintained stable conductivity under strain due to its excellent stretchability and mechanical robustness, minimizing interference with carbon paths resistance measurements.

To acquire silent speech signal, the sensor array was attached to face after removing the release paper from the adhesive surface. The transparent and stretchable substrate minimized visual interference and discomfort. A custom-designed flexible data acquisition circuit based on Arduino was used to simultaneously record resistance changes from seven channels and wirelessly transmit the data to a computer via Bluetooth. When a subject mouthed “OK” without vocal cord vibration, distinct signals were captured across all seven channels, shown in Figure 3c. The repeated and consistent waveforms confirmed both measurement repeatability and the reversibility of crack formation.

For hand gesture signal acquisition, the sensor array was placed on the back of the palm, rather than on the fingers, to avoid interfering with hand movements. The high sensitivity of the sensor array allows it to acquire skin strain variation caused by finger bending without attaching to the fingers. The subject performed hand gestures starting from a naturally open hand posture to mimic dynamic transitions in real sign language. When forming an “OK” hand gesture, the channels corresponding to the thumb and index finger showed significant changes, as shown in Figure 3f. In addition to these two examples, signals from various other silent speech phrases and hand gestures were collected with multiple repetitions. These multi-channel signals contain rich information for recognition.

### 3.4. Hybrid Neural Network Recognition and VR Display

**Figure 4:**
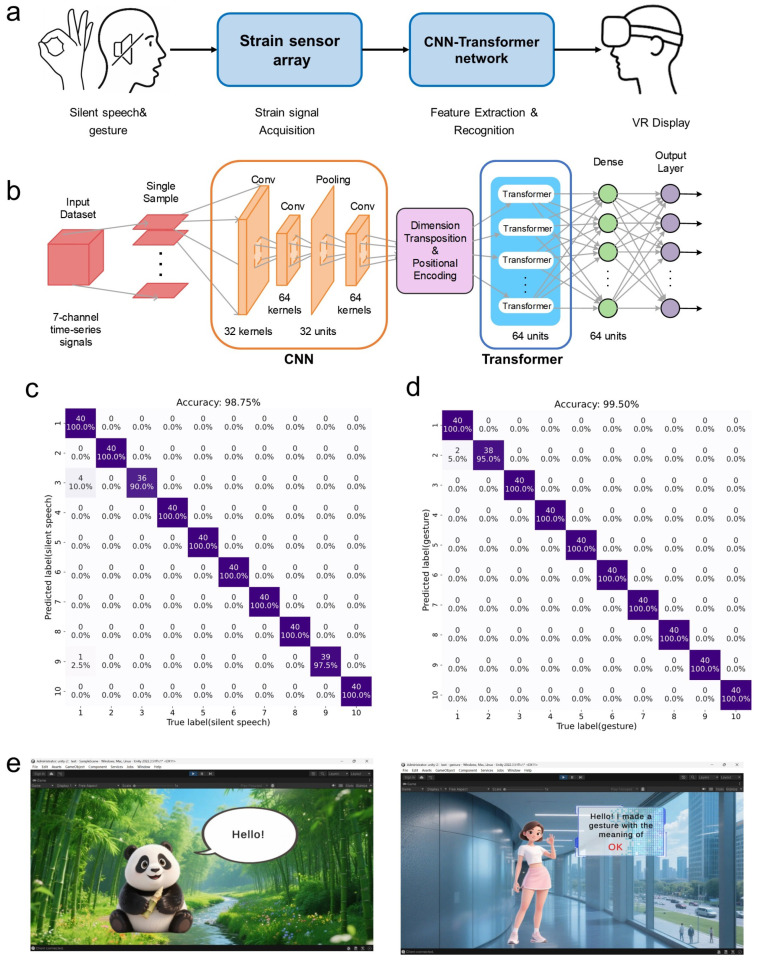
(**a**) Workflow of the silent-speech and hand gesture recognition system: signals are acquired by the strain-sensor arrays, processed by a hybrid neural network, and the recognized outputs are displayed in VR. (**b**) Schematic of the hybrid neural network architecture, which combines CNN and Transformer modules to extract complementary hierarchical features and improve accuracy. (**c**) Confusion matrix for silent-speech recognition. Labels 1–10 correspond to: OK, welcome, hello, bye-bye, morning, good night, yes, no, thank you, and “Xi’an Jiaotong University” (in Mandarin). (**d**) Confusion matrix for hand gesture recognition. Labels 1–10 correspond to hand gestures representing the digits 1–9 and the “OK” hand gesture. (**e**) Visualization of recognized silent-speech and hand gesture results in VR for multimodal interaction.

The proposed system recognizes phrases and hand gestures from strain signals, achieving accuracies of 98.75% and 99.5%, respectively. The recognition results were further visualized in a virtual reality (VR) environment. The overall system workflow is shown in Figure 4a.

A hybrid neural network combining a convolutional neural network (CNN) and a Transformer was proposed to recognize phrases and hand gestures from strain signals. This sequential CNN–Transformer architecture is illustrated in Figure 4b. The signal sequences captured by the strain sensor array during human movements have the characteristics of multi-channel, time-series, and inter-channel correlation. Therefore, we first use CNN to suppress noise and extract local features as well as inter-channel features, and then further extract long-range global features through the self-attention mechanism of Transformer. The combination of CNN and Transformer enables the model to learn both local and global patterns, resulting in high classification performance, as shown in the confusion matrix in Figure 4c,d.

The hybrid neural network proposed in this work exhibits significant advantages over standalone network architectures. CNN-only models are constrained by their fixed receptive field, making them unable to effectively capture long-range temporal dependencies in time-series data; meanwhile, recurrent models (e.g., LSTM) suffer from inherently low parallel computing efficiency, particularly when processing the long signal sequences acquired from human motion monitoring. In contrast, our hybrid framework leverages the complementary strengths of the two structures: the CNN module realizes noise suppression and efficient extraction of local and inter-channel features, while the Transformer further captures long-range global temporal dependencies through its self-attention mechanism, thus enabling comprehensive and robust feature extraction for the target sensing signals.

The recognized phrases and hand gestures were transmitted to a Unity environment and visualized in a virtual reality (VR) space, demonstrating the potential of this system for multimodal interaction [Figure 4e]. The neural network model files used in this study are available in the Supporting Information.

## 4. Discussion

### 4.1. Sensitivity and Range

Crack-based strain sensors often face a trade-off between sensing range and sensitivity. High sensitivity typically requires the conductive layer to crack easily under strain, causing a rapid increase in resistance. However, such conductive layer is prone to forming large cracks that can break the conductive path entirely at low strain levels, limiting the sensing range [26,27,28]. To address this issue, this study employs electrospinning to guide the formation of controlled microcracks over a wide strain range of 0–40% strain. The electrospun fibrous surface concentrates stress to promote small, distributed cracks, while enhancing adhesion to prevent delamination and large cracks that break the conductive path.

Electrospinning offers stretchability, self-assembly, and fine surface morphology. However, electrospinning requires a strictly limited flow rate to maintain the balance between electrostatic and surface tension forces, reducing efficiency. Forming a mechanically robust fibrous film often requires hours of deposition, making it unsuitable for large-area applications like epidermal devices.

To address this limitation, we identified a key insight: in crack-based sensors, only the surface morphology is functionally critical—bulk fiber thickness is unnecessary. While increasing fiber production per hour remains challenging, the required fiber amount per unit area can be significantly reduced. This work experimentally demonstrates that the electrospun layer obtained with only 5 min of electrospinning achieves performance comparable to a thick free-standing electrospun membrane produced over 4 h, and is sufficient for effective surface modification. However, using more advanced equipment to further reduce the electrospinning time and thickness, and exploring the critical thickness for efficient surface modification, will help further reduce costs and improve fabrication efficiency. The device fabrication in this work is based on small-scale manual preparation in the laboratory. In the future, employing large-scale manufacturing processes such as roll-to-roll production may help improve device uniformity and further reduce costs.

In this study, a thin electrospun layer is applied to commercial PU films (fabricated via casting), serving as a surface modification rather than a structural component. Additionally, screen printing enables high-throughput fabrication of multiple sensing channels simultaneously, supporting large-area signal acquisition and improving scalability for epidermal applications. Table 2 compares this work with similar strain sensors with promising epidermal applications. Compared with existing studies, this work adopts a high-efficiency fabrication process and easily accessible materials, while achieving comparable sensing performance. Moreover, this work further broadens its application prospects through innovations in hardware (flexible DAQ circuit) and algorithm.

### 4.2. Reducing Communication Barriers

This study aims to provide a more accessible communication method for individuals with voice disorders. From an informational perspective, human communication involves encoding and decoding messages—spoken language encodes thoughts into sound, which the listener then decodes into meaning [35,36]. Alternative methods such as sign language require both the speaker and the listener to learn new encoding and decoding systems, increasing the cognitive and social burden on both sides. In contrast, individuals with vocal cord disorders can still perform natural facial and oral movements associated with speech, without needing to learn a new encoding method. This form of expression is known as silent speech.

The proposed sensor system, combined with sequential CNN–Transformer architecture, captures epidermal strain signals generated by silent speech and decodes them into textual phrases. This study seeks to reduce communication barriers for people with speech disorders by translating natural, non-vocal expressions into VR interaction through epidermal strain sensing and AI-based recognition.

### 4.3. Limitations and Future Work

In this study, a flexible strain sensor was developed and preliminarily validated for silent speech and hand gesture recognition. However, due to constraints in available personnel and time, the current recognition experiments remain at an early stage. Future work should involve collaboration with clinical teams to recruit multiple participants, expand the vocabulary and hand gesture set, thereby optimizing sensor array performance and enhancing generalization capabilities.

Studies on crack density, crack spacing, stress concentration and the corresponding mechanisms are of great importance. However, due to the complex multiphase interface between electrospun fibers and conductive materials, relevant research is still insufficient and deserves further in-depth exploration.

Integration with other epidermal devices is feasible, as the fabrication process employed is highly compatible with printing epidermal electronics manufacturing methods [37,38,39]. The influence of temperature and humidity on the sensing performance of wearable devices is of great significance. However, owing to the absence of an in situ characterization setup that can simultaneously control temperature, humidity and strain, relevant experiments were not performed in this work. Future studies could explore the integration of this approach with surface electromyography (sEMG) or other epidermal sensing technologies on a single device to capture multi-modal physiological signals, thereby creating broader possibilities for diverse applications.

## 5. Conclusions

In summary, this work proposes a strain sensor based on electrospun surface modification and applies it to silent-speech and hand gesture recognition with VR interaction. By modifying the surface of a commercial PU film with a thin electrospun fibrous layer, we induce controlled microcrack formation in carbon-based screen-printed conductive traces, achieving high sensitivity with a gauge factor up to 243 within the 0–40% strain range. When configured as seven-channel arrays on the face and hand, the acquired strain signals can be decoded by a CNN–Transformer architecture (98.75% accuracy for silent speech and 99.5% for hand gesture recognition) and rendered in real time in VR environments to enable intuitive human–computer interaction. Notably, depositing only a thin electrospun layer on the substrate surface yields performance comparable to free-standing fibrous substrates while significantly reducing deposition time and material consumption, and it is compatible with screen printing to support scalable manufacturing. Although application-oriented research is still at an early stage, we hope this study will pave the way for the widespread use of electrospun sensors and, in the future, advance human well-being.

## Figures and Tables

**Table 1:** Performance comparison of strain sensors under three experimental conditions in this work.

Substrate	Sensing Material	Gauge Factor	Sensing Range	Electrospinning Time
Commercial PU	Carbon ink	Unstable & non-functional	Unstable & non-functional	0
Thick free-standing electrospun PU	Carbon ink	248	0–40%	4 h
Commercial PU + thin electrospun PU overlayer	Carbon ink	243	0–40%	5 min

**Table 2:** Performance Comparison of Various Stretchable Strain Sensors.

Substrate	Sensing Material	GF	Range	Electrospun	Arrayed, DAQ and AI	Durability	Ref.
Electrospun PVDF	MXene/PPy, etching + in-situ polymerization	78–355	0–100%	Long-term	NA	1000	[29]
Dragon Skin elastomer	Pt film/AgNW; sputtering + spray embedding	29–493	0–75%	NA	NA	1000	[30]
UV-ozone-treated PDMS	rGO/GO assembled at liquid–liquid interface	800–16,000	0–1%	NA	NA	6000	[31]
Electrospun SEBS	EGaIn liquid metal	4.423	0–100%	0.5 mm thickness	5-channel; integrated DAQ; CNN	2000	[32]
PDMS	CNF from pyrolyzed electrospun PAN	NA	Bending angle: 0–90^∘^	30 min	5-channel; integrated DAQ; SNN	NA	[33]
Electrospun TPU	AgNWs/TPU composite, solution spin-coating + in situ compounding	8	0–30%	Long-term	3-channel; NN classification algorithm	3000	[34]
Commercial PU + Electrospun PU	Screen-printed commercial carbon ink	243	0–40%	5 min	7-channel; flexible DAQ; CNN + Transformer	1000	This work

## Data Availability

The original contributions presented in this study are included in the article/Supplementary Materials. Further inquiries can be directed to the corresponding authors.

## Supplementary Materials

The following supporting information can be downloaded at https://www.mdpi.com/article/10.3390/nano16090520/s1, The configuration profile of the hybrid neural network used for silent-speech and hand gesture recognition is provided in the Supplementary Material.
