# Supplementary material for: Electrospun Surface-Modified Epidermal Strain Sensors Enable Silent Speech and Hand Gesture Recognition for Virtual Reality Interaction
Source: Nanomaterials (Basel). 2026 Apr 25;16(9):520. doi: 10.3390/nano16090520 (PMC13164596; doi:10.3390/nano16090520)
Supplement: Supplementary file 1 [file nanomaterials-16-00520-s001.zip › nanomaterials-4159537-supplementary.pdf]

{
″model_class″: ″SensorTransformer″,
″init_params″: {
″num_classes″: 10,
″input_dim″: 7,
″seq_len″: 512,
″d_model″: 64,
″nhead″: 8,
″num_layers″: 4
},
″architecture″: {
″conv″: ″nn.Sequential(″,
″nn.Conv1d(7, 32, kernel_size=5, padding=2), ″ ″nn.BatchNorm1d(32), ″,
″nn.GELU(), ″ ″nn.Conv1d(32, 64, kernel_size=3, padding=1), ″,
″nn.BatchNorm1d(64), ″ ″nn.GELU(), ″,
″nn.MaxPool1d(kernel_size=2), ″ ″nn.Conv1d(64, 64, kernel_size=3, padding=1), ″,
″nn.BatchNorm1d(64), ″ ″nn.GELU())″,
″pos_encoder″: ″nn.Parameter(torch.zeros(1, 256, 64))″,
″transformer″: ″nn.TransformerEncoder(″,
″nn.TransformerEncoderLayer(d_model=64, nhead=8, dim_feedforward=256, activation=’gelu’, batch_first=True), ″ ″num_layers=4)″,
″dense″: ″nn.Sequential(nn.Linear(64, 64), nn.GELU())″,

″classifier″: ″nn.Linear(64, 10)″

},

″note″: ″7-channel input → Conv (32 kernels) → Conv (64 kernels) → Pooling → Conv (64 kernels) → Dimension Transposition & Positional Encoding → Transformer (64 units) → Dense (64 units) → Output Layer (10 classes).″

}
